# Sex between men in the context of HIV: The AIDS 2008 Jonathan Mann Memorial Lecture in health and human rights

**DOI:** 10.1186/1758-2652-11-9

**Published:** 2008-12-24

**Authors:** Jorge Saavedra, Jose Antonio Izazola-Licea, Chris Beyrer

**Affiliations:** 1 National AIDS Program, (CENSIDA), Mexico City, Mexico; 2 AIDS Financing and Economics Division, Joint United Nations Programme on HIV/AIDS (UNAIDS), Geneva, Switzerland; 3 Center for Public Health and Human Rights, Johns Hopkins Bloomberg School of Public Health, Baltimore, MD, USA

## Abstract

Gay, bisexual, and other men who have sex with men (MSM) have been among the most affected populations by HIV since the AIDS pandemic was first identified in the 1980s. Evidence from a wide range of studies show that these men remain at the highest risk for HIV acquisition in both developed and developing countries, and that despite three decades of evidence of their vulnerability to HIV, they remain under-served and under-studied. Prevention strategies targeted to MSM are markedly under-funded in most countries, leading to limited access to health services including prevention, treatment, and care. We explore the global epidemic among MSM in 2008, the limited funding available globally to respond to these epidemics, and the human rights contexts and factors which drive HIV spread and limit HIV responses for these men.

What do we mean by the term MSM? MSM is a construct from the 1990s that tries to capture behavior and not identity. It was crafted to avoid stigmatizing and culturally laden terms such as gay or bisexual, which do not capture the wide diversity of orientations, sexual practices, cultures, and contextual settings in which male same-sex behaviors occur, and where HIV transmission and acquisition risks are centered. MSM includes both gay and non-gay identified men, bisexual men, and MSM who identify themselves as heterosexuals. It also includes men engaging in "situational" sex between men, such as can occur in prisons, schools, militaries or other environments; and it includes male sex workers who may be of any orientation but are often at very high risk for HIV. MSM may include some biologically male transgender persons, though some do not identify as male. And MSM includes a wide array of traditional and local terms worldwide–with enormous cultural diversity in Asia, Africa, Latin America and elsewhere. We use the term MSM here at its most inclusive.

## Review

### Epidemiology of HIV among MSM

While cultural and human rights contexts, as well as prejudice, misconceptions and ignorance, are clearly important for understanding HIV/AIDS among MSM, risks for acquisition and transmission are grounded in individual behavior. At the individual level there are a number of known risk factors for HIV infection among MSM. These include unprotected anal intercourse (UAI), with the greatest risk being for receptive UAI; a high frequency of male partners (>3 per week); high numbers of lifetime male partners (>10); and a history of injection drug use (IDU)–these are individuals with dual MSM and IDU risk, and we see these where IDU and MSM epidemics overlap. There also is emerging evidence of the role of non injection drugs, especially methamphetamines, which likely increase sexual risks for MSM. In the United States, Black/African American race is associated with much higher odds of being HIV infected even when controlling for such factors as socio-economic status and educational level [5].

**Figure 1:**
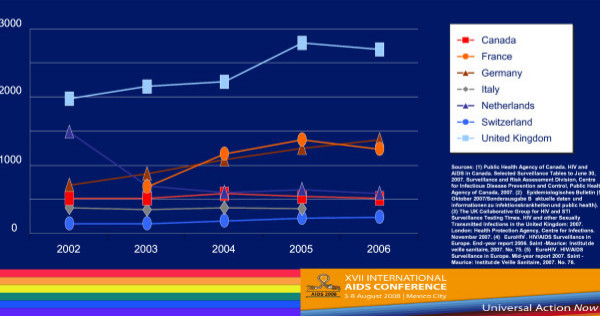
**HIV infections newly diagnosed in men who have sex with men, by country, and year of report, 2002–2006**. UNAIDS 2008 Global Report.

**Figure 2:**
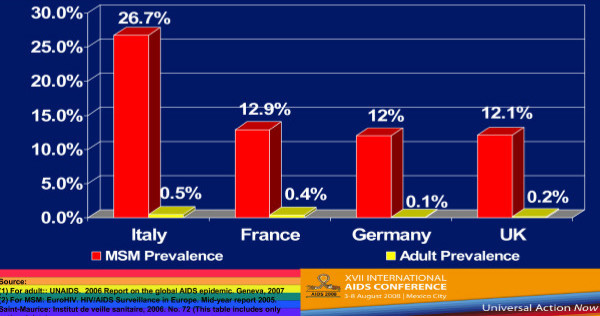
**HIV Prevalence rates in 4 EU Countries: MSM and General Adult prevalence rates**.

**Figure 3:**
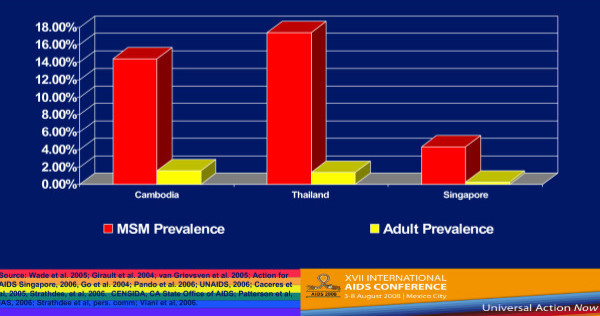
**HIV Prevalence among MSM and general populations in selected Southeast Asian countries**.

**Figure 4:**
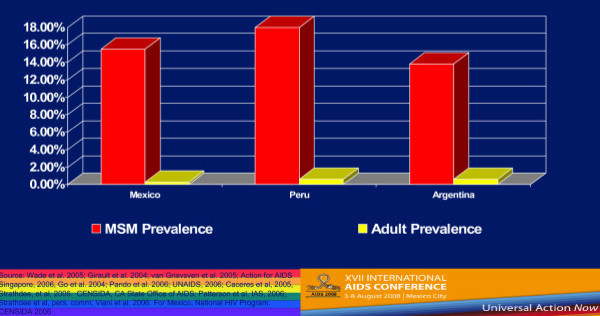
**HIV Prevalence among MSM and general populations in selected Latin-American countries**.

**Figure 5:**
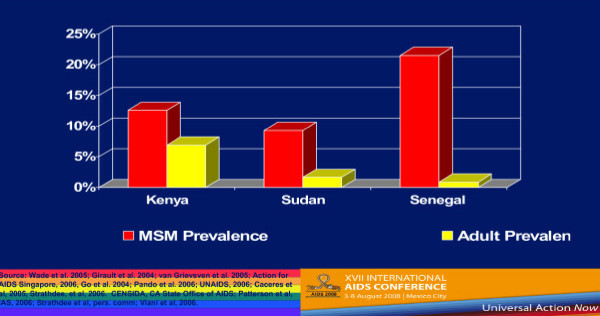
**HIV Prevalence among MSM and general populations in selected African countries**.

Country-level population estimates for HIV among MSM reveal a number of trends. Figure 1 shows 2002–2006 UNAIDS data on HIV rates among MSM from Western Europe and Canada–the trends are generally either stable or increasing [4]. But if we compare MSM HIV infection rates and the rates of HIV among the general population of reproductive age adults in selected countries in Western Europe, we see that MSM are at much greater risk for HIV infection (Figure 2) [4-6]. These much higher levels of HIV prevalence among MSM are seen in Southeast Asia (Figure 3) [7-9], as well as in Latin America, where we see much higher rates of HIV among MSM in contrast to reproductive age adults in Mexico, Peru, and Argentina (Figure 4) [10-12]. This epidemiologic scenario holds true even in generalized epidemics in the world's most HIV affected region, Sub-Saharan Africa (Figure 5) [13-15]. African MSM populations are among the most hidden and stigmatized worldwide, and the data are sparse, but nevertheless, this same epidemiologic pattern holds.

**Figure 6:**
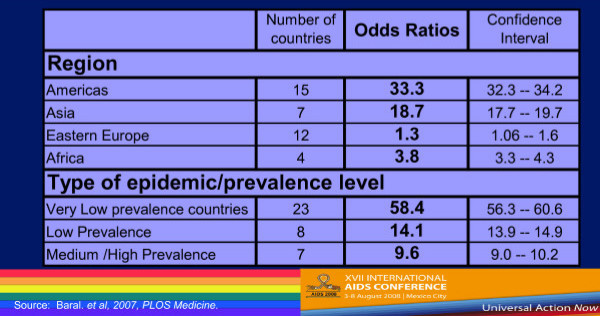
**Systematic Review of HIV among MSM compared to reproductive age adults prevalence: adjusted odds ratios by region and prevalence level**.

To further explore this global trend, Baral, et al, conducted a systematic review of the global literature from 2000–2006 and calculated adjusted odds ratios for MSM compared to reproductive age adults by region and by prevalence (Figure 6)[16]. In Latin America, MSM were 33 times more likely to have HIV infection; in Asia, more than 18 times; and in Africa, 3.8 times as likely. These odds ratios were statistically robust despite the fact that data were available from only 38 low- and middle-income countries, and from only 4 African studies. Whenever HIV prevalence studies have been done among MSM the same consistent results appear. Yet, global responses have not been commensurate to these realities. MSM remain under-studied, under-served, under-funded and frequently ignored or denied by governments. We must ask why.

### MSM, HIV, and human rights

A number of factors that influence risk and vulnerability for MSM are contextual–they impact individual men in complex ways, but they are fundamentally social and structural realities. These include human rights violations, the criminalization of sexual orientation and same-sex behavior, and social stigma. UNAIDS has recognized that realizing human rights is an essential component of success in responding to HIV/AIDS: "Long-term success in responding to the epidemic will require sustained progress in reducing human rights violations associated with it, including gender inequality, stigma and discrimination." While this statement does not explicitly address MSM, it certainly includes them.

How do rights limitations affect MSM vulnerability to HIV infection "on the ground?"

Most obviously, where same-sex behavior is criminalized, MSM remain hidden, they will be very cautious or refuse to get an HIV test, even if they feel they need it, and those seeking to reach them with services, from condoms and lubricants to education and treatment outreach, can be harassed for supporting illegal activities. A recent example is from Nepal, where police have beaten peer outreach workers for attempting to distribute condoms. In the Indian situation as well, police have used the fact that same-sex behavior is illegal to harass outreach workers and interrupt prevention activities. This is the case despite the fact that India's National AIDS Control Organization (NACO), which receives substantial government funding, has been strongly supportive of MSM prevention activities In fact, NACO has filed a court challenge to India's sodomy laws on the basis that they are a barrier to HIV prevention. (Personal communication from Sujata Rao (08/05/08). Head of NACO (National AIDS Control Program of India).

Another recent example of state-sanctioned homophobia impacting HIV programs for MSM comes from Uganda. At the recent PEPFAR Implementers meeting in Kampala, Uganda, three LGBT activists were arrested and charged while peacefully demonstrating for access to HIV services for MSM. On 25 July, 2008, the Ugandan police arrested and tortured one of the three (the only man) for his continued call for access to health services [17].

These are not isolated incidents, unfortunately, but examples of the continued limitation on the rights to freedom of expression, privacy, and the rights of consenting adults to choose their sex and life partners. The international lesbian and gay association, ILGA, maintains a website with the status of rights and protections worldwide [18]. Eighty-six countries in the world criminalize sex between consenting adult men, and more than half of all African countries do so. Nine States have death penalties for homosexual relations between consenting adults including: Iran, Mauritania, Nigeria, Pakistan, Saudi Arabia, Somalia, Sudan, United Arab Emirates, and Yemen (laws in existence in 2004).

For example, in Senegal the law states that: "Without prejudice to the more serious penalties provided for in the preceding paragraphs or by articles 320 and 321 of this Code, whoever will have committed an improper or unnatural act with a person of the same sex will be punished by imprisonment of between one and five years and by a fine of 100,000 to 1,500,000 francs. If the act was committed with a person below the age of 21, the maximum penalty will always be applied."

From Senegal, is the following personal communication to one of the authors (JS): " I am HIV positive man. Here, in my country, to be gay is against the law. The ones who are, like myself, we cannot say it openly. In order to socialize or meet other gay people, or to dance and be with our boyfriends, we need to go to clandestine places, these places are frequently closed by the police or city authorities, therefore every three months we need to look for the new one. When we go out of the clandestine gay bar, most of us will separate and take different roads to our own homes, in order to return to "normal" life, with our wives and kids. The only possible way to have a socially acceptable life, here in Senegal is to marry and have children" Personal communication with Jorge Saavedra by JOPH, person living with HIV in Dakar (December, 10, 2003).

How do these laws compare to international human rights standards? The International Convention on the Eradication of All Forms of Discrimination Against Women (CEDAW) states that: "Nobody should be forced to marry against their will and people should have the right to choose who they marry." Surely this basic right to choose one's spouse, which was gained by the women's movement, should apply equally to men, including when their choice is a same sex spouse.

The XVII International AIDS Conference began with the world's first international march against homophobia. In advance of the march one of the authors (JS) alerted LGBT activists and the government of Panama that the march would highlight Panama as the only Latin American country still criminalizing homosexuality. That law was changed by executive order two days before the march, making Latin America the first region of the once called the third world, to be completely free of these discriminatory laws. (Belize and Guyana, although they are part of the American continent, are not Latin countries, and are generally grouped as part of the Caribbean.)

As part of honoring Dr. Jonathan Mann, who pioneered work on the interface of HIV and human rights, the HIV/AIDS community needs to address the human rights protections for MSM and other sexual minorities. The International Covenant on Civil and Political Rights (ICCPR) guarantees human rights "without distinction of any kind, such as race, color, sex, language, religion, political or other opinion, national or social origin, property, birth or other status" [19]. In 1994 the Human Rights Committee held that, under the ICCPR, sexual orientation was a status protected from discrimination, with reference to *"sex" *including *"sexual orientation." *This ruling has made clear that discrimination based on sexual orientation is a fundamental human rights violation. Realizing this right, and ending discrimination, will likely be essential to achieving universal access to HIV prevention, treatment, and care for MSM. This is a key part of the response, particularly in those settings where social stigma and isolation for MSM remain the most marked.

### The response: Marginalization of MSM in the global response to HIV/AIDS

**Figure 7:**
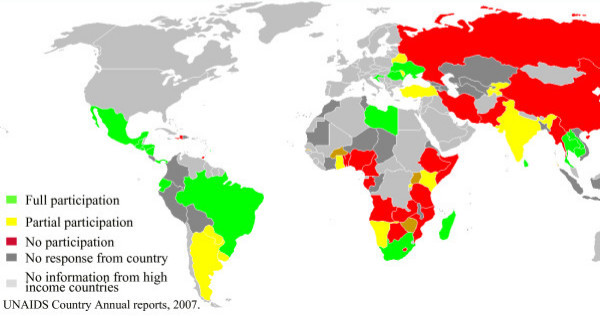
**National Planning – Level of participation of the organistions representing MSM in national AIDS reviews in 38 low and middle income countries**.

There is evidence of the marginalization of MSM in the global response from the United Nations Joint Program on AIDS (UNAIDS) annual survey conducted among the UNAIDS country coordinators in low- and middle-income countries [20]. Indeed, when probed about the level of participation of the organizations representing MSM in national AIDS planning and reviews, most countries did not even report on MSM participation (Figure 7) [20]. Full participation was reported in 24% of these countries; in 15% there was "insufficient, yet increasing participation". However, this global average blurs important geographical differences. For example, the lowest level of participation reported was in West and Central Africa, where 0% reported full participation of MSM organizations, only 8% of countries reported having "insufficient, yet increasing participation", and 15% reported "insufficient participation with no sign of improvement". The region reporting the highest level of participation was Latin America, with 50% of countries reporting full participation and 19% reporting "insufficient, yet increasing participation."

The reports cited above would seem to warrant some optimism given that a reported 72% of national AIDS action frameworks included prevention programs for MSM. However, this finding seems to be at odds with other results from the same survey. For example, only 31% of national reports on the epidemic produced globally in 2007 included government publication of "a surveillance report that includes MSM". There was only one country in sub-Saharan Africa that included such a report, and one country in the Middle East and North Africa region that did so. While half of the countries in the Asia Pacific region reported that they had a government surveillance report disaggregating HIV or AIDS data for MSM in 2007, only 38% of Latin American countries and 25% of Caribbean countries had such a report.

This lack of participation has been matched by very limited funding for MSM-specific programs. United Nations (UN) Member States are required to submit country progress reports to the UNAIDS Secretariat every two years to monitor progress towards the goals established in the UN General Assembly's (UNGASS) 2001 Declaration of Commitment on HIV/AIDS (DoC). In adopting the 2001 Declaration of Commitment on HIV/AIDS, UN Member States obligated themselves to regularly report on their progress to the General Assembly. The Secretary-General charged the UNAIDS Secretariat with the responsibility for developing the reporting process, accepting reports from member States on his behalf, and preparing a regular report for the General Assembly. These progress reports include data on HIV spending (by activity and source) as the first indicator of national commitment. This indicator measures total HIV expenditures, as well as separate expenditures for HIV prevention (and other seven other groups of services and programs). The specific expenditure targeting MSM is to be disaggregated from of the sub-total for prevention [21].

Unfortunately, not all countries have been able to report with precision their disaggregated expenditures. A total of 109 country reports included HIV expenditure information for 2006 [4]. However, of this total, there were only 55 low- and middle-income countries where the preventive expenditure could be disaggregated by population. The disaggregated data indicate that a mere 0.6% of prevention expenditures were actually spent on targeted prevention for MSM.

How do the expenditures relate to the needs? A preliminary analysis of country reports on MSM expenditures, as reported for the monitoring of the Declaration of Commitment (DoC) signed during the Special Session of the United Nations General Assembly in 2001 (UNGASS 2001), could be compared with the 2006 country resource need estimates that UNAIDS previously developed for its report on Global Resource Needs 2006–2008 [22] using the National AIDS Spending Assessments (NASA) classification [23]. Such an analysis demonstrates the existence of a substantial financing gap (defined as the difference between the resource needs and the actual spending) in thirty-eight (38) countries with data on MSM financing, indicating that while there was an estimated financial need of US $29 million in 2006 (assuming 80% coverage of an essential prevention package among the population of MSM in each country), the expenditure in these countries was estimated at just US $3 million. (The selection of the 38 countries for the comparison between resource needs and expenditures in 2006 was based on the availability of data at the time of the XVII International AIDS Conference in Mexico in August 2008. The expenditure reports are owned by reporting countries. The resource needs estimates have not been validated by country officials.)

**Figure 8:**
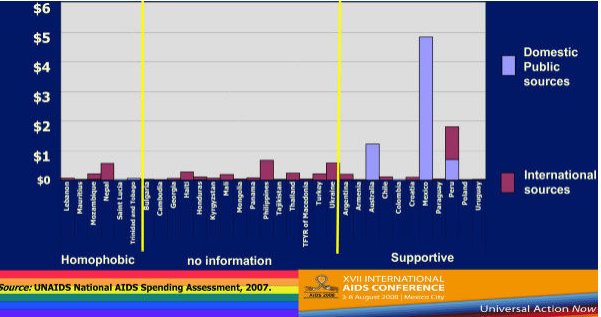
**Reported expenditures for programs specifically aimed at/involving men who have sex with men (million USD)**.

Figure 8 shows data on the reported prevention expenditures aimed at MSM in this subset of 38 countries–and this is unacceptably low [23]. Even if slightly different from the larger set of countries presented above, in this subset of countries, just 1.2% of prevention expenditures were aimed at MSM, with 1% aimed at commercial sex workers, and 2% at harm reduction programs and injecting drug users. The remaining 96% of prevention expenditures were dedicated to non-targeted prevention efforts. Preventive expenditures on MSM in countries with generalized epidemics accounts for 0.1% of total prevention efforts (or US $0.001 per inhabitant), while in countries with low level epidemics it represents 2.9% of prevention spending (or US $0.004 per inhabitant), and in concentrated epidemics, 3% (or US $0.01 per inhabitant).

### What are MSM receiving as preventive services?

Based on country reports to monitor progress in the implementation of the DoC, the percentage of MSM in 27 countries who report knowing where they could receive an HIV test, and who received condoms between 2005 and 2007 was 40%. Although this was an improvement, since only 9% of MSM responded similarly just two years earlier. Nevertheless, this very basic and limited measure of access to services still is extremely low.

### What is the effect of stigma and discrimination on preventing, or in failing to prevent, HIV transmission?

Countries where non-discrimination laws and regulations exist show higher coverage rates of prevention services among MSM. The median percentage of MSM who reported receiving condoms and knowing where they could be tested was almost 60% in countries with protective laws or regulations, compared with countries that do not have such policies (38%) [4].

When countries are classified according to the ILGA classification of the policy environment with respect to MSM [18], there is a directionally indicative correlation between higher expenditures from in-country resources and MSM supportive policies. Expenditures are lower or not reported in countries classified as having homophobic policy environments.

### What is needed for a more complete response?

Both individual and structural level actions are called for. A minimum package of services would include: education, behavioral interventions, peer outreach, condom promotion and social marketing, lubricants, HIV voluntary counseling and testing, sexually transmitted infections diagnosis and treatment, antiretroviral treatment, and access to care and support. Addressing homophobia and providing MSM-friendly services in health services would also be required, along with efforts to reduce the social-structural determinants of HIV risk and vulnerability for these men including decriminalization, addressing stigma, discrimination and homophobia, and making concrete advances in human rights. This requires funding commensurate with need.

**Figure 9:**
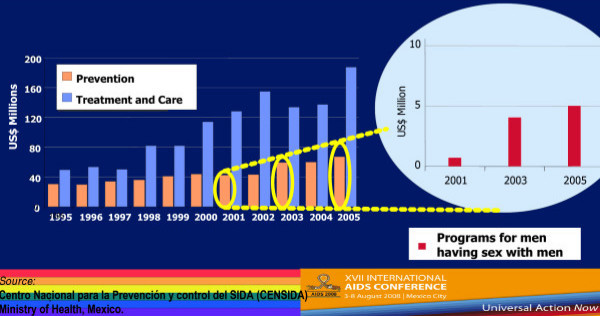
**HIV spending on prevention, treatment and care, Mexico, 1995–2005 and detailed spending for programs specifically aimed at men who have sex with men**.

In the National AIDS Program of Mexico, expenditures for HIV have increased overall from 2001 to 2005. They have also expanded for MSM specific programs (Figure 9). This is both good public policy and good evidence-based decision making, since MSM remain by far the most at-risk group in Mexico [10].

## Conclusion

HIV continues to disproportionately affect MSM worldwide. The exclusion of MSM from surveillance, targeted prevention, and treatment and care still limits the global response to HIV/AIDS. To improve the human rights and health of MSM comprehensive advocacy efforts are needed. In the third decade of AIDS it is time to realize the equal rights of MSM to access health and other services in environments that are friendly to their sexual orientation, and to recognize that meeting these needs is both a global public health priority, and a compelling human rights issue. To achieve the latter, there is a clear need to significantly increase the proportion of HIV resources targeted towards MSM.

This paper derives from the plenary speech of the same name, presented during the XVII International AIDS Conference (AIDS 2008). AIDS 2008 was perhaps the most comprehensive and intensive International AIDS Conference focusing on the realities of MSM and HIV globally. The events began with the first International March Against Homophobia. In the Opening Session of the Conference UN Secretary-General Ban Ki-moon stated: "I urge nations to pass laws against homophobia". Immediately afterwards, both the President and Minister of Health of Mexico addressed directly and openly the need to eradicate homophobia. The aftermath has been far reaching: before the finalization of this paper, one of the authors (CB) was notified that, because of the denouncement of the violation of the rights of MSM activists in Uganda during this plenary, the charges against the detained activists were dropped by the authorities [24].

The XVII International AIDS Conference was a milestone, where the MSM community, along with policy- and decision-makers, said: Enough! Evidence must inform policies. The epidemiological evidence is striking; it has existed for a long time; and we cannot continue failing to respond. Despite the occasional mention in national plans, the inadequate response to MSM has been demonstrated by low coverage rates of basic preventive services for these men, and by the scarcity of targeted spending, –still reaching only one tenth of the estimated resource needs. Such data underscore the urgent need to act now to stop homophobia, stigma and discrimination in order to deliver quality and effective services to MSM. When countries, international agencies and donors begin to target their efforts and resources based on the epidemiological evidence, and adjust funding levels to be commensurate with the HIV burden among MSM, we will see dramatic changes in the response to HIV in multiple settings. Funding efficiency can only increase when flows are directed towards populations with the greatest unmet HIV prevention needs.

## Competing interests

The authors declare that they have no competing interests.

Jose Antonio Izazola-Licea was an employee of the Joint United Nations Programme on HIV/AIDS (UNAIDS) as chief of the AIDS Financing and Economics Division at the time of the writing of this paper. His co-authorship does not necessarily represent the institutional view of UNAIDS.

## Authors' contributions

JS conceived the plenary presentation and manuscript. CB led the epidemiology component, and JAIL led the unmet needs analsis. All reviewed the literature and contributed to the writing of the manuscript. All authors read and approved the final manuscript.
